# The extent of intrauterine growth restriction determines the severity of cerebral injury and neurobehavioural deficits in rodents

**DOI:** 10.1371/journal.pone.0184653

**Published:** 2017-09-21

**Authors:** Crystal A. Ruff, Stuart D. Faulkner, Prakasham Rumajogee, Stephanie Beldick, Warren Foltz, Jennifer Corrigan, Alfred Basilious, Shangjun Jiang, Shanojan Thiyagalingam, Jerome Y. Yager, Michael G. Fehlings

**Affiliations:** 1 Division of Genetics and Development, Krembil Research Institute, Toronto, Ontario, Canada; 2 Institute of Medical Science, University of Toronto, Toronto, Ontario, Canada; 3 Faculty of Medicine, University of Toronto, Toronto, Ontario, Canada; 4 STARR facility, Toronto Medical Discovery Tower, Toronto, Ontario, Canada; 5 Section of Pediatric Neurosciences, Department of Pediatrics, University of Alberta, Edmonton, Alberta, Canada; 6 Division of Neurosurgery, University of Toronto, Toronto, Ontario, Canada; Hopital Robert Debre, FRANCE

## Abstract

**Background:**

Cerebral Palsy (CP) is the most common physical pediatric neurodevelopmental disorder and spastic diplegic injury is its most frequent subtype. CP results in substantial neuromotor and cognitive impairments that have significant socioeconomic impact. Despite this, its underlying pathophysiological mechanisms and etiology remain incompletely understood. Furthermore, there is a need for clinically relevant injury models, which a) reflect the heterogeneity of the condition and b) can be used to evaluate new translational therapies. To address these key knowledge gaps, we characterized a chronic placental insufficiency (PI) model, using bilateral uterine artery ligation (BUAL) of dams. This injury model results in intrauterine growth restriction (IUGR) in pups, and animals recapitulate the human phenotype both in terms of neurobehavioural and anatomical deficits.

**Methods:**

Effects of BUAL were studied using luxol fast blue (LFB)/hematoxylin & eosin (H&E) staining, immunohistochemistry, quantitative Magnetic Resonance Imaging (MRI), and Catwalk neurobehavioural tests.

**Results:**

Neuroanatomical analysis revealed regional ventricular enlargement and corpus callosum thinning in IUGR animals, which was correlated with the extent of growth restriction. Olig2 staining revealed reductions in oligodendrocyte density in white and grey matter structures, including the corpus callosum, optic chiasm, and nucleus accumbens. The caudate nucleus, along with other brain structures such as the optic chiasm, internal capsule, septofimbrial and lateral septal nuclei, exhibited reduced size in animals with IUGR. The size of the pretectal nucleus was reduced only in moderately injured animals. MAG/NF200 staining demonstrated reduced myelination and axonal counts in the corpus callosum of IUGR animals. NeuN staining revealed changes in neuronal density in the hippocampus and in the thickness of hippocampal CA2 and CA3 regions. Diffusion weighted imaging (DWI) revealed regional white and grey matter changes at 3 weeks of age. Furthermore, neurobehavioural testing demonstrated neuromotor impairments in animals with IUGR in paw intensities, swing speed, relative print positions, and phase dispersions.

**Conclusions:**

We have characterized a rodent model of IUGR and have demonstrated that the neuroanatomical and neurobehavioural deficits mirror the severity of the IUGR injury. This model has the potential to be applied to examine the pathobiology of and potential therapeutic strategies for IUGR-related brain injury. Thus, this work has potential translational relevance for the study of CP.

## Introduction

Encephalopathy during early life is one of the major causes of lifelong neurological disability. Abnormalities during pregnancy and damage to the brain during fetal development can have severe consequences, including preterm birth—leading to encephalopathy of prematurity (EoP)—and subsequent diagnoses of neurological conditions later in life such as epilepsy, cognitive delay, neuro-sensory deficits and cerebral palsy (CP) [1]. Of these disorders, CP is the most common pediatric neurodevelopmental physical disability, occurring in about 2.0–3.5/1000 live births [2]. This number increases as birthweight decreases, with children born at very low birth weights (<1500 g) exhibiting CP in 50-72/1000 live births [3,4]. The risk of developing CP has indeed been strongly linked to growth restriction and birth weight [5,6], and it is one of the only disorders whose incidence has not decreased as the quality of healthcare has increased over the past forty years [7,8]. As a lifelong, non-progressive condition, the associated additional healthcare and support costs per child with CP have been estimated at roughly 1.5 million dollars [9], reaching 8.2 billion total expenditure per year in developed countries [10].

Phenotypically, the majority of children with CP present with bilateral spasticity (52%) [3,11]. Spastic quadriplegia and diplegia are the clinical phenotype associated with oligodendroglial injury in newborns, termed periventricular leukomalacia (PVL) [12]. PVL is the most common form of brain injury in premature infants, due to the susceptibility of the periventricular white matter (WM) to focal ischemic and inflammatory destructive processes. The oligodendrocyte (OL) is intrinsically vulnerable to the neurotoxic effects of glutamate and other excitatory amino acids in a maturation dependent fashion [13–15]. The most vulnerable stage of OL development is that of the late-stage premature OLs (preOLs) [16–19]. There is also evidence of grey matter involvement in clinical CP [20–23], accounting for those children with significant accompanying cognitive, sensory, and epileptic co-morbidities.

Chronic placental insufficiency (PI)—a lack of sufficient delivery of nutrients to the developing fetus—is one of the leading causative factors for EoP and CP, resulting in intrauterine growth restriction (IUGR), ischemic neurological injury, and the development of neuromotor symptoms in over 60% of cases [24,25]. Following periods of *in utero* hypo-perfusion, preOL-specific cell depletion occurs in periventricular watershed regions (proximal to descending corticospinal tracts). This damage often leads to bilateral spastic CP diagnoses in early postnatal life [12,26]. Clinically, CP presents as a spectrum, with increasing neurological involvement proportional to the severity of neurological injury [27–29].

Currently, treatment for early life brain damage and CP is restricted to rescue, rehabilitative, and orthopedic approaches–there being no preventive or regenerative biological therapies for this condition. Moreover, the translation of pre-clinical therapeutic approaches has met with difficult challenges. In this regard, the development of pre-clinical models replicating the human condition will allow for a better understanding of the underlying disease state, and provide a platform through which the potential of biologic therapeutics can be evaluated.

Given the complexity and far-reaching implications of EoP, and specifically CP, it is clear that further research is needed to unravel the causes of, mechanisms of, and potential interventions for this disease. Currently, IUGR models mainly exist in mice, rats, rabbits, and guinea pigs [30]. These animal models show WM injury in different regions of the brain, which mirror the main clinical symptoms of EoP and CP, including behavior and cognition [31]. While the use of these models has helped to elucidate the underlying mechanisms that lead to PI-induced brain damage, there is a shortage of long-term animal studies that have utilized a multipronged approach consisting of MRI, behavioural, and histological outcome measures. As EoP and CP result in chronic disability, it is important to evaluate these IUGR models over extended periods of time using a variety of assessment tools, which will allow for more relevant testing and translation of therapeutics to the clinic. In this article, we have characterized the spectrum of phenotypic differences in the rat IUGR model for at least 16 weeks after birth, describing subtle differences between mildly and moderately growth-restricted animals. These animals could be compared to very low birth weight (VLBW) and extremely low birth weight (ELBW) humans, respectively, who are classified as having EoP and often go on to develop CP. We chose to assess various regions of the brain that can be affected and lead to phenotypic deficits following encephalopathy. Our major focus was on WM tracts, as individuals who have experienced EoP or CP caused by fetal brain damage commonly exhibit demyelination and damaged OLs [32,33]. We also focused on some grey matter regions [34] like the hippocampus and caudate, which when damaged, lead to an array of cognitive and physical disabilities [35]. Recent systematic reviews by our group showed that neuronal cell death, white matter injury, and motor and cognitive deficits mimic the abnormalities observed in CP after hypoxia-Ischemia [15] and IUGR [30], verifying that uterine artery ligation is a clinically relevant model.

Thus, in the present paper, we aim to develop and validate a clinically relevant rodent model of PI that demonstrates the differential spectrum of disease outcomes (described herein), which can be used to accurately and reproducibly induce IUGR injuries which a) exhibit unique behavioural, gross anatomical (MRI), and cellular changes that comprise the disease phenotype and b) are specifically targetable by novel translational treatment interventions, such as cell therapies, as described previously [36,37].

## Materials and methods

### Breeding and animal husbandry

A total of 29 Long Evans (LE) rats (Charles River, Sherbrooke CA) of either sex, were used in this study. Rats were sourced from an inbred colony maintained at the University of Alberta facility. They were maintained on a normal 12-hour light/dark cycle and had access to food and water *ad libitum*. All experimental protocols were approved by the animal care committee of the University Health Network, Toronto, and the University of Alberta, Edmonton, in accordance with the Canadian Council on Animal Care Guidelines. Groups were defined according to birth weight. Normal birth weight for our Long Evans rat pups was 6.27±0.37g. For this study, we classified pups born 2 SDs below the mean (<5.52g at birth) as mild IUGR (n = 5, birth weight = 5.40 g ± 0.07), and 4 SDs below the mean (<4.76g) as moderate IUGR (n = 9, birth weight = 3.79 g ± 0.14). Sham animals (n = 12) had an average birth weight of 6.35 g ± 0.11. Two sham rats and one IUGR rat died prior to the end of the study due to respiratory infections.

### IUGR surgery

Pregnant female rats were anesthetized with Isoflurane at E20 (embryonic day 20) and underwent bilateral uterine artery ligation (BUAL), in order to induce PI and IUGR, as previously described [38]. A midline abdominal incision was created to access the uterine horn and the uterine arteries were bilaterally ligated with 5–0 silk sutures (S1 Fig). Sham operations in which the arteries were not ligated were performed on the dams of control animals. Muscle layers were sutured together using absorbable sutures (3–0, catgut), and the skin was closed using 5–0 silk sutures. Dams were given buprenorphine analgesic (0.05–0.1 mg/kg) and observed every 2–3 hours thereafter. Pups were delivered spontaneously at term (E23). Offspring weight was recorded at birth and those animals that were more than 2 standard deviations (SD) below mean birth weight (<5.52g) were considered growth restricted. For this study, IUGR animals were further classified into mild (2–4 SD below mean) and moderate (>4 SD below mean) injury groups. The surgeries resulted in 12 Sham, 5 mild IUGR, and 9 moderate IUGR animals.

### MRI acquisition parameters

MRI was performed in a 7T horizontal magnet (Bruker Biospec 70/30, Ettlingen, Germany) with water-cooled B-GA12 gradient insert with a 7.2 cm inner diameter radiofrequency (RF) coil. A volume resonator was used for RF transmission with an actively decoupled surface coil for RF reception. During scanning animals were anaesthetized with 5% isoflurane with oxygen (1.5–2% thereafter for maintenance), and placed prone on a heated animal bed. Water at 37°C was flushed through inlaid pipes in the bed in order to maintain body temperature at 37°C. The head was fixed in a stereotactic frame with a tooth bar, nose cone and head restraint. Respiration rate (40-60bpm) was monitored and maintained continuously during scanning (SA instruments, New York). Animals were imaged at 3 weeks of age.

Coronal T2 weighted images were obtained with either a 2D turbo spin (RARE) sequence (TE = 72 ms, TR = 4800 ms, MTX = 200x200, resolution = 0.15 x 0.15 x 0.8 mm, rare factor = 24, 3 averages), or 3D RARE sequence (TE = 71 ms, TR = 1800 ms, MTX = 192x192x 48, resolution = 0.15 x 0.15 x 0.4 mm, rare factor 24). Due to advancements in imaging technologies during the course of the study some of the animals were imaged using a 2D acquisition method while others used a 3D acquisition method. These differences did not affect the ability to draw ROIs and calculate relevant values for the animals.

Diffusion-weighted imaging (DWI) was performed with either a 2D respiratory-gated EPI sequence (TE = 36 ms, TR determined by respiratory interval ~ 1300 ms, MTX = 200x200, resolution = 0.150 x 0.150 x 0.8 mm using 6 directions [39] and b value = 750 s/mm^2^; 1 b0 image), or 3D respiratory gated EPI sequence (TE = 39 ms, TR determined by respiratory interval ~ 1300, MTX = 192x192x 48, resolution = 0.15 x 0.15 x 0.4 mm, 12 directions (Jones et al., 1999) and b value = 1500 s/mm^2^; 1 b0 image). 6 coronal slices were selected from each brain for analysis, covering the entire corpus callosum (CC). The use of both 2D and 3D acquisitions were due to advancements in imaging technologies during the course of the study as discussed above.

### MRI data analysis

DW images were processed as follows: Three eigenvalues (λ_1_, λ_2_, and λ_3_) and eigenvectors were calculated for each voxel of each image using Diffusion Toolkit software (version 0.6.2.2). Fractional anisotropy (FA), axial (λ_1_) and radial [(λ_2_ + λ_3_)/2] diffusivities, along with all associated calculations were generated using this software. ROIs were drawn bilaterally around the genu (gCC), body (bCC), and splenium of the corpus callosum (sCC), as well as around the external capsule (Ec), and caudate (Ca). The prefixes “g”, “b”, and “s” indicate that these locations were drawn at the level of the gCC, bCC, and sCC, respectively. ROIs were defined using MIPAV software (version 5.3.4) utilizing a combination of FA and FA color maps. These were cross-checked with co-registered T2 maps to ensure accuracy of anatomical placement. ROI data from each animal were assessed for left-right hemisphere differences. Since no inter-hemispheric differences were found, data were combined for both hemispheres and the means (±SEM) for each group are presented.

### Tissue processing

Following the last behavioral testing time-point at 16 weeks of age, rats were terminally anaesthetized with isoflurane and intracardially perfused with cold phosphate-buffered saline (PBS) and cold 4% pH 7.4 paraformaldehyde (PFA). Whole brains were isolated and post-fixed in 10% sucrose in 4% PFA overnight and then cryoprotected with 30% sucrose in PBS for at least 24 hours. Brains were embedded in Optical Cutting Temperature Compound (OCT, Tissue-Tek/Sakura Finetek—Fisher Scientific, Ottawa, CA). Serial coronal tissue sections were cut at 40μm on a Leica cryostat throughout the entire corpus callosum, collecting three sections per slide.

### Histology

Histological processing was performed on every tenth slide. Luxol Fast Blue (LFB)/Hemotoxylin and Eosin (H&E) staining was performed according to methods well established in our laboratory [40]. Immunohistochemistry was performed according to Ruff and colleagues [41]. The following primary antibody concentrations were used: rabbit anti-Olig2 1:500 (Millipore, AB9610), mouse anti-NeuN 1:400 (Millipore, MAB377), mouse anti-MAG 1:350 (Millipore, MAB1567), and rabbit anti-NF200 1:500 (Sigma, N4142). Secondary antibodies were diluted to 1:300 concentrations. Alexa Fluor® 488 Goat Anti-Mouse IgG Antibody (A11031) was used as a secondary antibody for MAG and NeuN. Alexa Fluor® 568 Goat Anti-Rabbit IgG Antibody (A11011) was used as a secondary antibody for Olig2 and NF200.

Immunohistochemical images in the corpus callosum were taken at five predetermined regions in each section. Images in the hippocampus were taken within three predetermined regions per hippocampus—within the CA1, CA2, CA3, and dentate gyrus in each section (S2 Fig). Quantification of each cell surface marker was performed in each of these regions and averaged for each animal. Values from individual animals were further averaged within their respective experimental groups. Images were taken using a Zeiss LSM 500 microscope at 40x magnification (NeuN, Olig2) or 63x magnification (MAG, and NF200).

Whole-brain LFB/H&E images were taken at 4x magnification using the Stereoinvestigator program (MBF Bioscience, Williston, USA) and the Nikon Eclipse E800 microscope at five rostrocaudally-matched sections throughout the corpus callosum. Images were then analyzed using ImageJ software (http://imagej.nih.gov), and major brain structures (corpus callosum, hippocampi, fornix, fimbria, internal capsules, caudate nuclei, optic chiasm, nucleus accumbens, septofimbrial nuclei, pretectal nuclei, mammillothalamic fasciculus, and ventricles) were examined throughout this region. Corpus callosum and hippocampal thickness were measured at three sites per structure on each section (S3 Fig).

For immunohistochemical quantification, the number of Olig2+ cells within each 40x field (see S2 Fig) was counted per image for the corpus callosum. The average density of cells within each tissue sample was calculated from this for each animal, averaged within experimental groups, and then compared between groups. Similarly, the densities of neurons within the CA2 and CA3 regions of the hippocampus were counted and averaged separately for each animal, averaged within groups, and then compared between groups. Percent myelination was calculated by counting the total number of NF200+/MAG- (unmyelinated) axons and the number of NF200+/MAG+ (myelinated) axons per 63x field, and then calculating the percentage of NF200+/MAG- axons out of the total number of axons counted (myelinated and unmyelinated). Olig2+ cells in other brain structures were assessed in each designated 63x region. Thickness and area of the CA2, CA3, and dentate gyrus were measured using ImageJ.

### Behavioural testing using catwalk gait analysis

Rats were tested using Catwalk gait analysis software (Noldus, CatWalk 7.1, Ottawa, CA) to assess neurobehavioural deficits. Rats were familiarized with the equipment 2–3 days prior to testing. Animals were run through a clear Plexiglas elevated platform that was enclosed to make a tunnel; paw placement, as well as gait behaviour, were recorded by a camera located underneath. Three uninterrupted passages from one end to the other were analyzed and averaged per rat at each timepoint. Analysis was performed on a minimum of 3 successive uninterrupted step cycles *a priori*, using qualitatively the best 3 runs for analysis. Multiple parameters were evaluated, including: paw intensity (measure of paw pressure generated during placement), swing speed (measure of the velocity of the moving limb during the swing phase of the step cycle), print position (measure of the relative print position of the hind paw compared to the ipsilateral front paw), and phase dispersion (a measure of inter-limb coordination). Animals were tested at 3, 6, 9, 12 and 16 weeks of age.

### Statistical analysis

All analyses were performed using a one-way analysis of variance (ANOVA), followed by Tukey’s post-hoc test. A two-way ANOVA followed by Tukey’s post-hoc test was performed for the Catwalk gait analysis and the tracking of weight over time. Significance is indicated as follows: *p<0.05; **p<0.01; ***p<0.001. Error bars in all graphs represent standard error of the mean (SEM).

## Results

### BUAL leads to growth restriction and changes in overall brain structure

**Fig 1:**
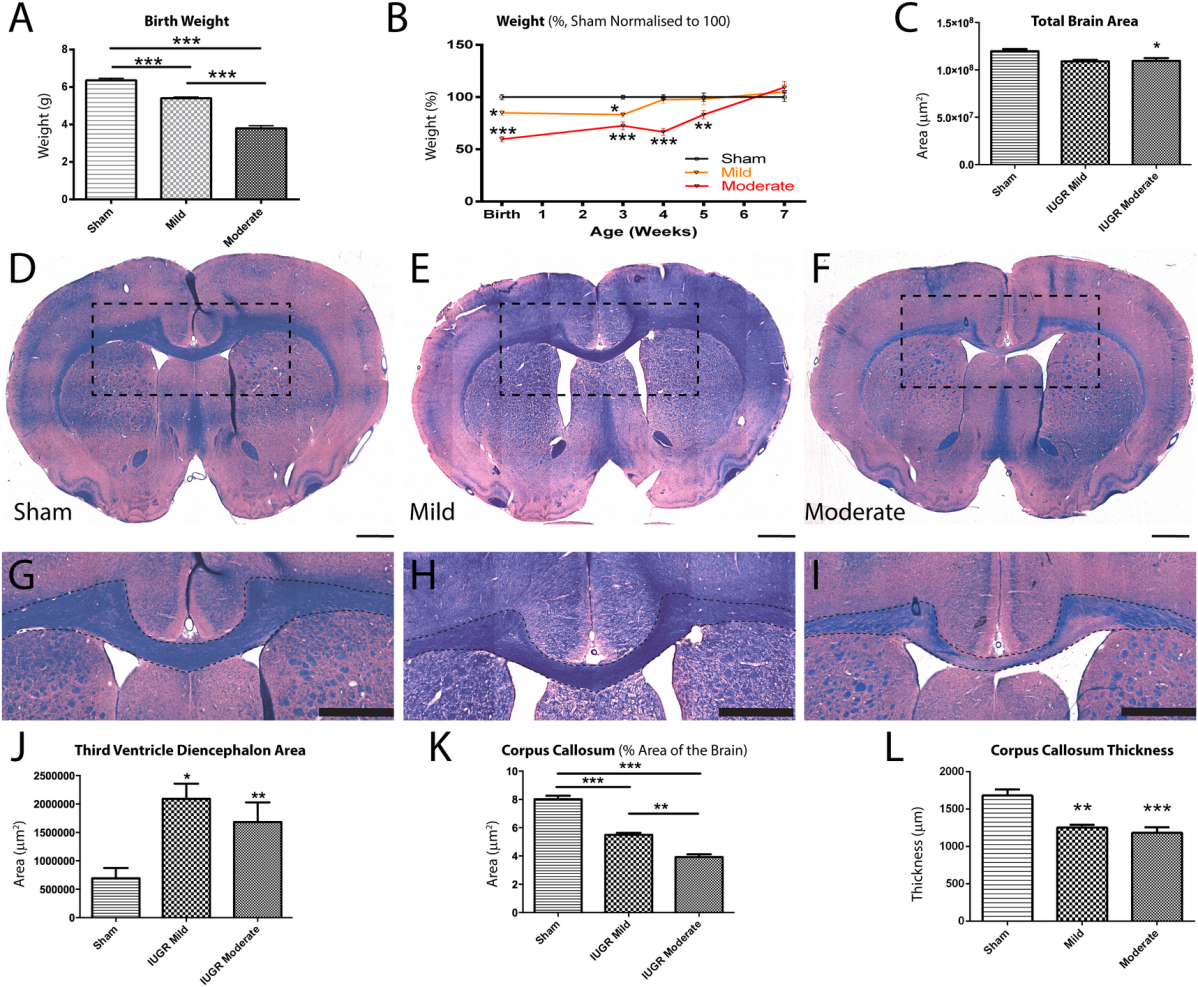
Whole brain, third ventricle and corpus callosum structural abnormalities in the brains of IUGR animals. Animals were initially separated into different severities of IUGR based on birth weight; moderate IUGR animals had significantly lower birthweights than sham controls and mild IUGR animals, while mild IUGR only had significantly lower birthweight than sham controls (A). Although weight eventually normalized in adulthood (B), animals classified to have moderate IUGR at birth displayed persistent decreases in total brain area in adulthood, though this was not the case for mild IUGR animals (C). Representative whole-brain sections are depicted in (D-F) for sham, mild IUGR, and moderate IUGR, respectively. The CC is delineated in (G-I) for sham, mild IUGR, and moderate IUGR, respectively. Both groups of IUGR animals also displayed significant ventricular enlargement at the level of the diencephalon when compared to sham controls (J). The area of the CC was reduced in IUGR animals when compared to sham controls, and the CC of moderate IUGR animals was significantly smaller than mild IUGR animals (K). The thickness of the CC was also significantly reduced in both mild and moderate IUGR groups when compared to sham animals (L). Scale Bar = 1000 μm; *p<0.05; **p<0.01; ***p<0.001.

To assess the effect of BUAL on the extent of growth restriction, animals were categorized into mild and moderate cohorts depending on their weights at birth, and compared to sham controls. The three groups all had significantly different weights from each other at birth (Fig 1A), though body weights normalized by 6 weeks (Fig 1B). To assess the integrity of brain structures after IUGR, LFB/H&E sections were imaged and areas were compared quantitatively using ImageJ software. IUGR animals exhibited reductions in adult brain area, which reached significance in moderately, but not mildly injured IUGR animals (Fig 1C–1F). Regardless of the extent of growth restriction at birth, we saw ventricular enlargement in all IUGR animals, which persisted into adulthood. This reached significance in the region of the diencephalon only (Fig 1J).

### Effect of IUGR on the corpus callosum

We then examined the corpus callosum (CC; Fig 1G–1I), one of the largest white matter tracts in the brain, and one that is often affected in patients with CP [42]. Taking into consideration that moderate IUGR animals exhibited smaller brain sizes as a baseline, when we investigated CC size as a percentage of total brain area, animals with greater growth restriction at birth exhibited smaller CC areas than less growth-restricted animals (Fig 1K). Thus, IUGR animals exhibited decreases in CC area, which was in addition to the decreased overall brain area discussed above. Lastly, CC thickness was also significantly reduced in both groups of injured animals when compared to controls (Fig 1L).

### Changes in axial and radial diffusivities in IUGR animals (Fig 2)

MRI is a common modality used to visualize and categorize injury in preclinical and clinical studies. We assessed WM integrity in various anatomically-relevant structures using DWI at 3 weeks of age (juvenile). Axial diffusivities were significantly increased in the gCC and sCC of mild IUGR animals when compared to sham animals (S1 Table). This was similar in moderate IUGR animals, though the increase at the location of the sCC was not significant. Importantly, moderate IUGR animals showed significant increases in radial diffusivity in the gCC, gEc, bEc, gCa and when compared to sham animals. This is indicative of subtle axonal and/or myelin injury in these anatomical regions. FA values were significantly decreased in the gCa of moderate IUGR animals when compared to sham animals. Surprisingly, FA was increased in the bCa and sCC of the moderate IUGR group. This could be explained by the fact that FA is a composite value of axial and radial diffusivities, and so subtle changes in one or both diffusivities could have led to these results.

**Fig 2:**
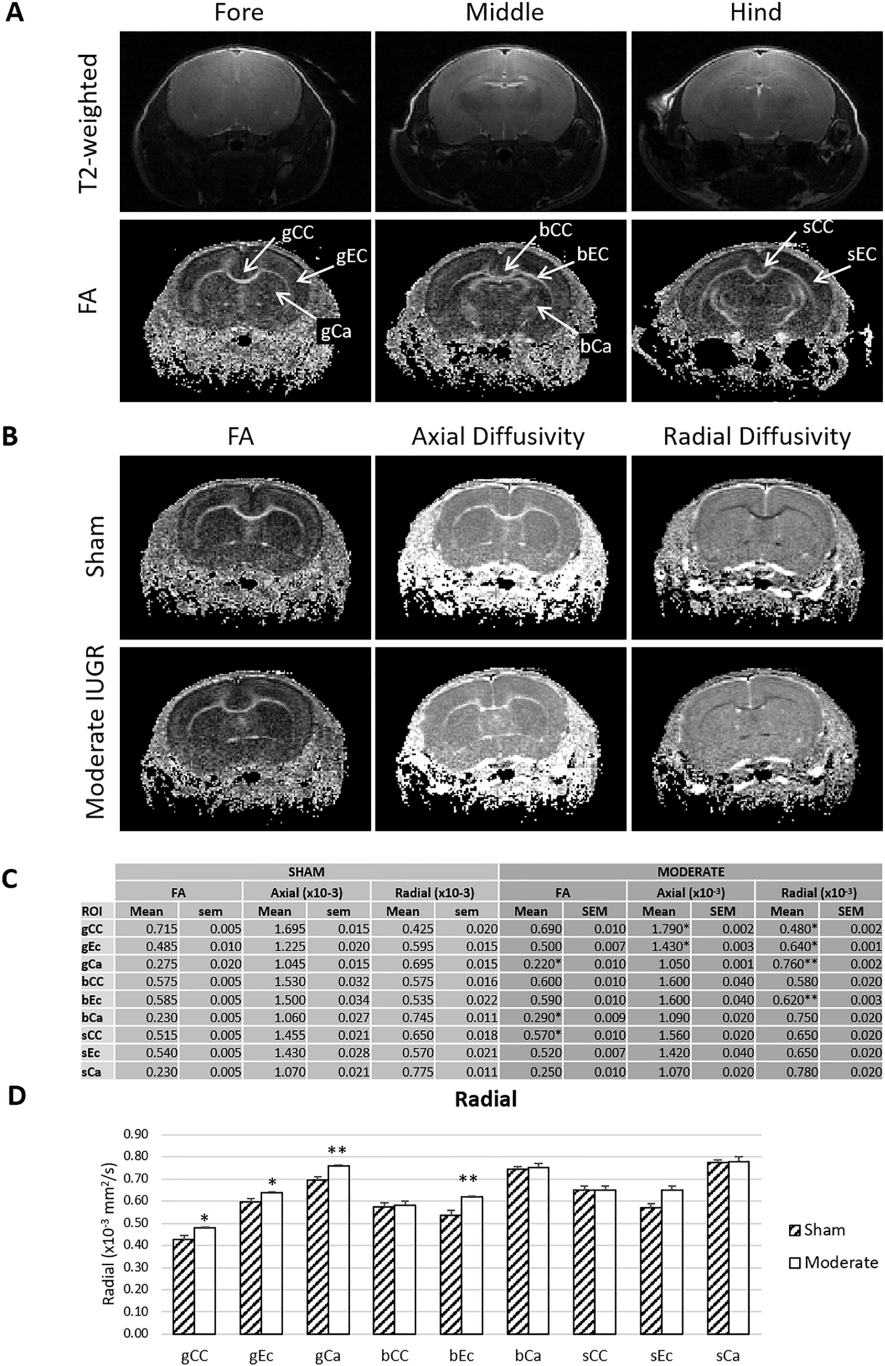
MRI reveals differences between moderate IUGR and sham animals. At 3 weeks, abnormalities in both axial and radial diffusivities in white and grey matter were evident in moderate IUGR animals. (A) Representative T2-weighted images and fractional anisotropy (FA) maps, highlighting locations of ROIs in the corpus callosum (CC), external capsule (EC), and caudate (CA) at three anatomical levels (g: genu; b: body; and s: splenium). (B) Representative FA, axial diffusivity, and radial diffusivity maps at the level of the genu from sham and moderate IUGR rats. Note the higher radial diffusivities in the CC of the moderate IUGR animal when compared to sham, with similar FA and axial diffusivities. (C) Tabulated DTI metrics across the sham and moderate IUGR cohorts. Locations of significant differences are highlighted by * (p<0.05) and ** (p<0.01). (D) Graphical display of radial diffusivities in each ROI location for the moderate injury cohort (*p<0.05; **p<0.01).

### White matter cell death in the corpus callosum varies with the extent of fetal growth restriction

**Fig 3:**
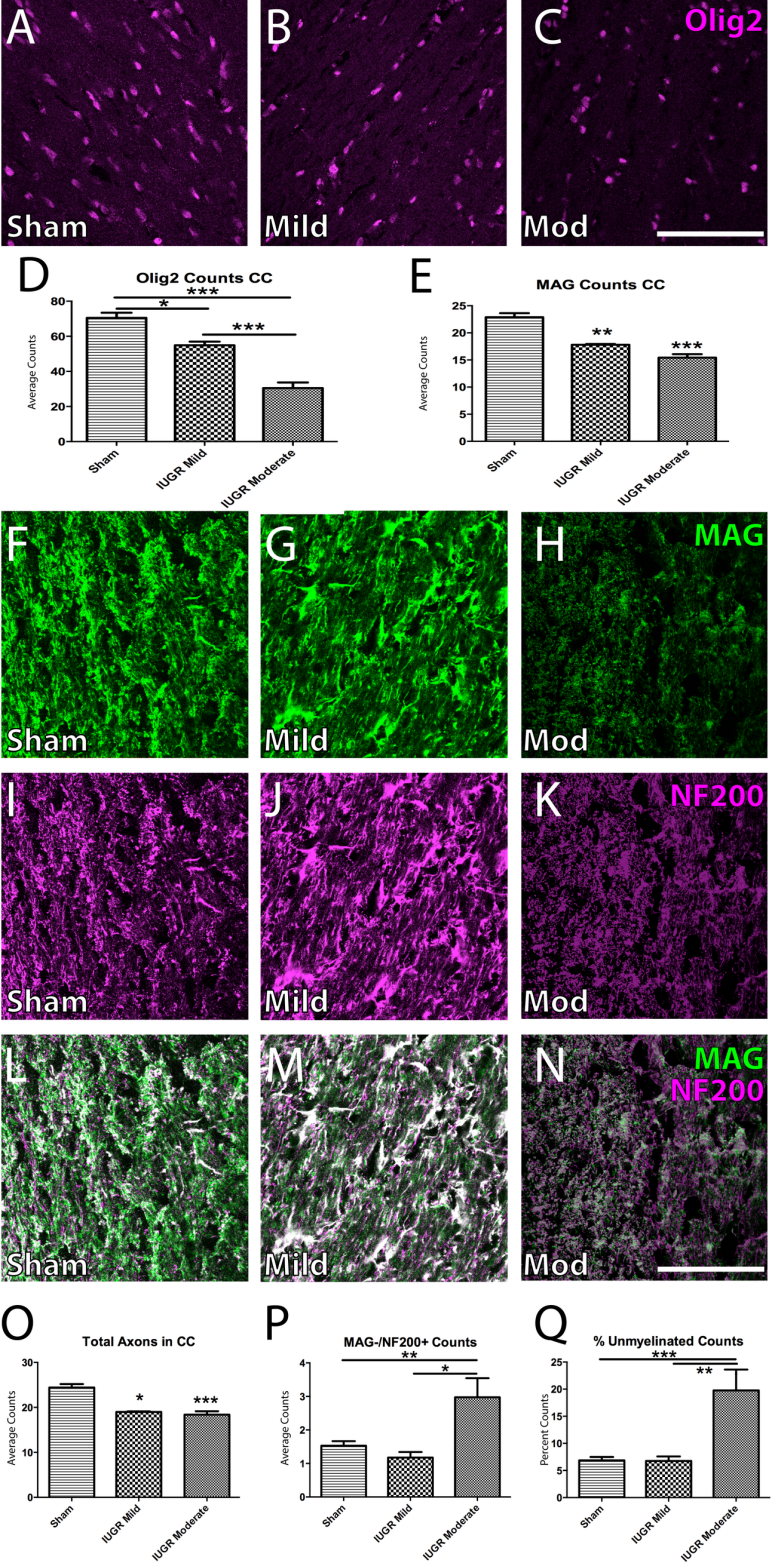
IUGR is accompanied by a loss of white matter in the corpus callosum. Olig2+ OL counts in the corpus callosum revealed a decrease in OLs in mild IUGR (B), and moderate IUGR (C) animals, but not in sham controls (A). This decrease was significantly different in both IUGR groups when compared to sham controls; moderate IUGR animals also displayed significantly reduced counts when compared to mild IUGR animals (D). Both mild and moderate IUGR animals had decreased myelination (as assessed through MAG+ staining) when compared to sham controls (F-H). The number of NF200+ axons was also reduced in both mild and moderate IUGR groups when compared to sham controls (I-K). Merged images of MAG+/NF200+ staining are also shown (L-N). There were fewer axons in IUGR animals regardless of the severity of injury (O). A significantly greater proportion of the remaining axons were unmyelinated (MAG-/NF200+) in only moderate IUGR animals, while mild IUGR animals exhibited a phenotype similar to sham controls (P). When we investigated the results in (P) as a percentage of total axons in the CC, only moderately injured animals had a significantly higher proportion of unmyelinated axons when compared to other groups (Q). Scale Bar = 100 μm;*p<0.05; **p<0.01; ***p<0.001.

Based on the histopathological changes we observed in the CC (Fig 1), we continued to investigate WM integrity using immunohistochemistry to examine numbers of Olig2+ OLs and MAG+ myelination of NF200+ axons. Within the CC, Olig2+ counts revealed decreasing OL density inversely proportional to the extent of growth restriction (Fig 3A–3D).

When we looked at myelination and axonal properties in the CC, we found an intermediate phenotype. There were lower absolute numbers of MAG+ myelinated axons in both mild and moderate IUGR animals (Fig 3E–3H). Likewise, when we calculated the total number of axons (myelinated NF200+/MAG+ and unmyelinated NF200+/MAG-), deficits were revealed in both IUGR groups, compared to sham (Fig 3I–3O). The absolute number of unmyelinated axons showed a difference from Sham animals only in moderate IUGR animals (Fig 3P). When normalized to the total number of axons, the percentage of unmyelinated fibres was unaffected in mild IUGR animals and was increased only in moderate IUGR animals (Fig 3Q). Despite having fewer OLs and axons, mildly affected animals had a similar percentage of myelinated axons as Shams. These results show that the amount of myelination in the CC is dependent upon the severity of IUGR and that total axonal counts are affected regardless of the severity of injury.

### Changes to the hippocampus following IUGR

**Fig 4:**
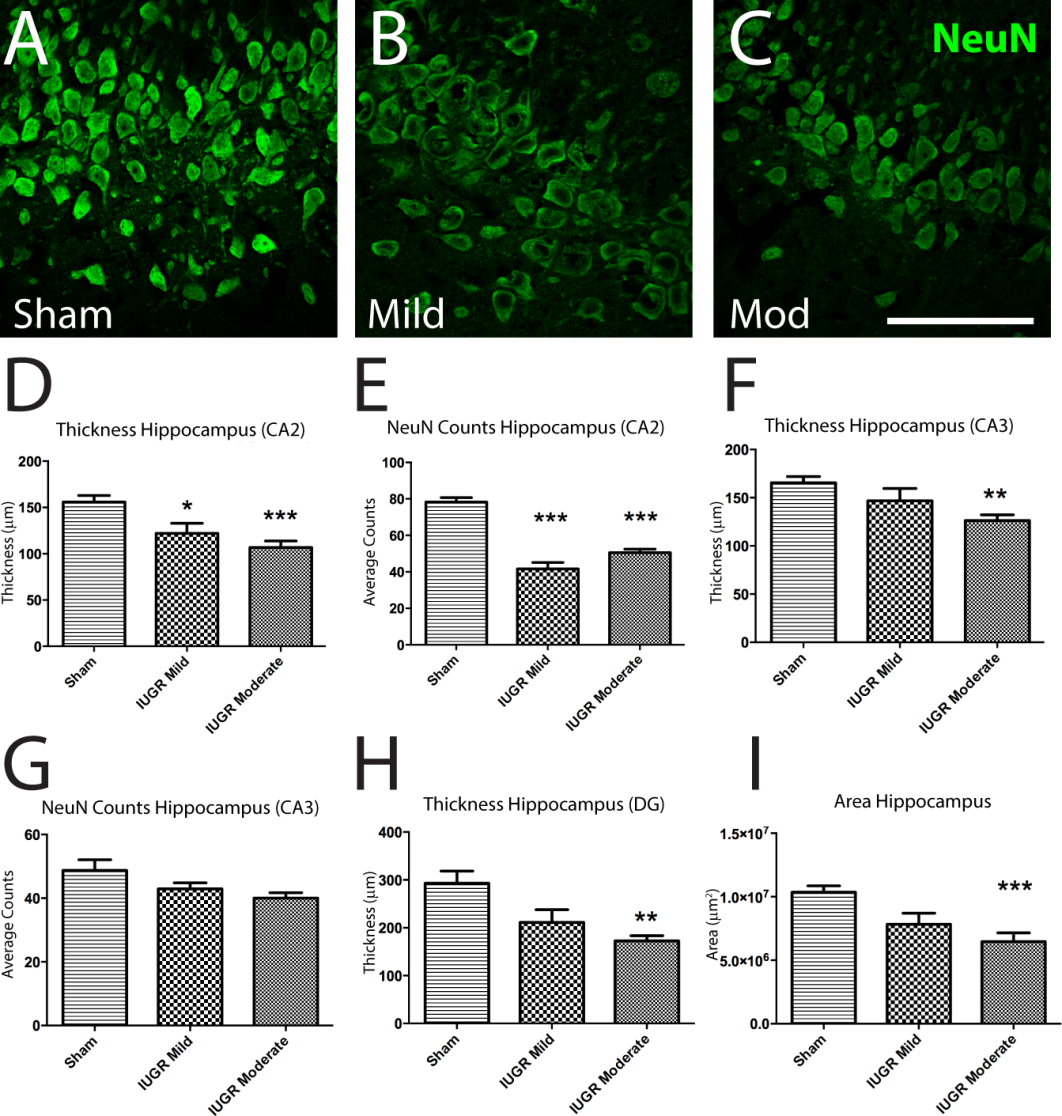
Hippocampal structure is differentially affected according to the severity of IUGR. NeuN+ staining in the hippocampus (A-C) revealed severity-specific deficits in CA2 (D-E), CA3 (F-G) and the dentate gyrus (H). Quantification of hippocampal area revealed a significantly reduced size only in moderate IUGR animals when compared to sham animals (I). Scale Bar = 1000 μm;*p<0.05; **p<0.01; ***p<0.001.

The hippocampus is an important grey matter structure that plays a key role in the processing and integration of various forms of memory. Damage to the hippocampus leads to cognitive deficits that can recapitulate some phenotypes seen in children with neurological conditions. Adult animals that experienced IUGR exhibited damage to hippocampal structures when compared to Sham surgery controls (Fig 4A–4C). When we examined the CA1 region, no consistent reductions in thickness and neuronal cell loss were observed. Both mild and moderate IUGR animals exhibited neuronal cell loss in CA2 (Fig 4D) and reduced numbers of NeuN+ neurons (Fig 4E). Thickness of the CA2 region was inversely correlated with the extent of IUGR, though this did not reach significance. When we examined the CA3 region, we saw decreased thickness only in moderate IUGR animals (Fig 4F), and NeuN counts were not significantly different among the three groups (Fig 4G). Similar to CA3, the thickness of the dentate gyrus showed a trend of decreasing thickness with increased growth restriction at birth, which reached significance only in moderately injured animals (Fig 4H). Furthermore, only moderate IUGR animals exhibited reductions in overall hippocampal area, when assessed on LFB/H&E-stained sections, (Fig 4I). The areas of the fornix and fimbria (Fornix: Sham vs. Mild p = 0.927, Sham vs. Moderate p = 0.919; Fimbria: Sham vs. Mild p = 0.061, Sham vs. Moderate p = 0.136) and Olig2+ cell counts in the fimbria (Sham vs. Mild p = 0.835, Sham vs. Moderate p = 0.211) were unaffected in both IUGR groups.

### Changes to the caudate nucleus following IUGR

**Fig 5:**
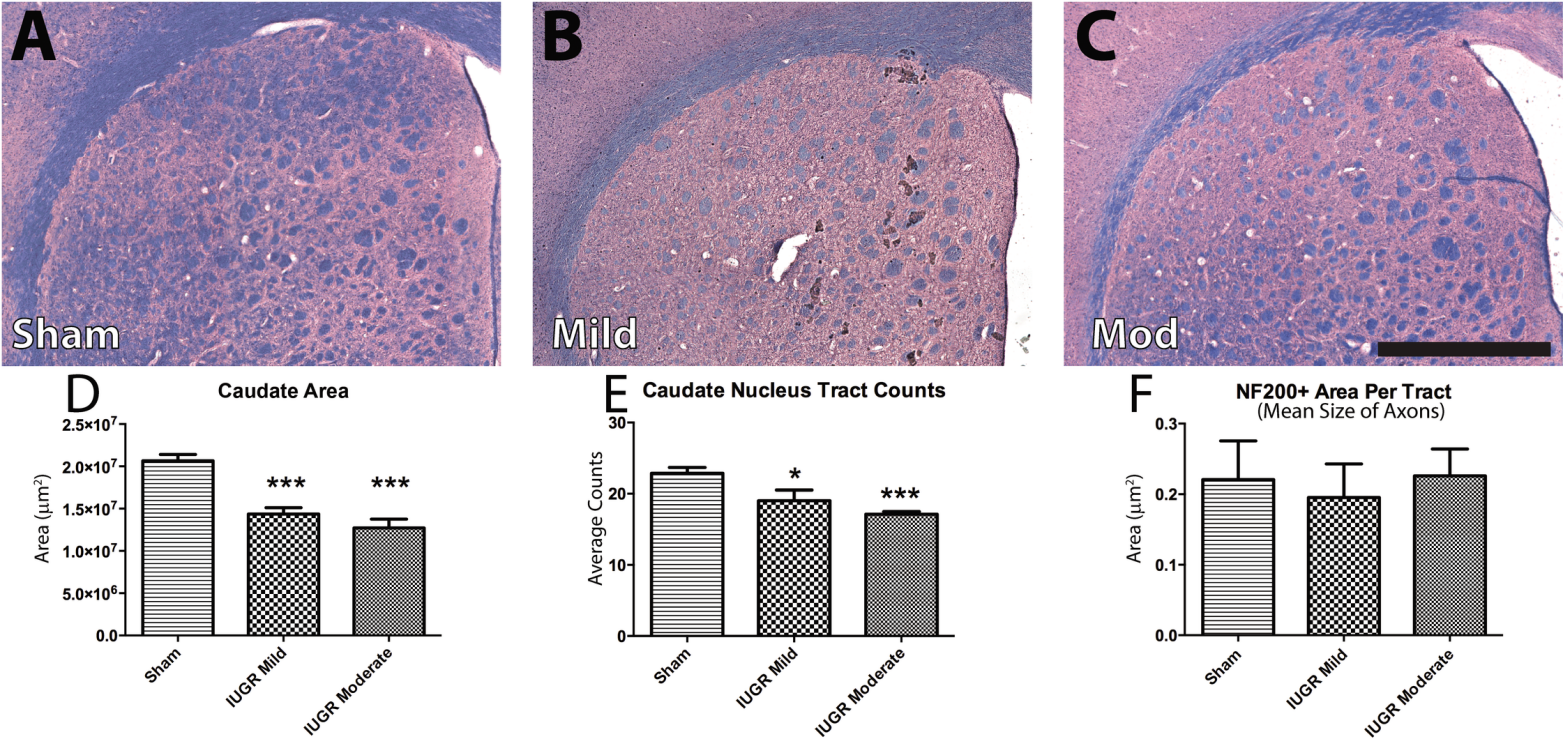
Reduced area and number of tracts in the caudate nucleus. The caudate nucleus was affected by IUGR, as depicted in representative LFB/H&E stains (A-C). This structure showed reduced size in IUGR animals, regardless of severity (D). IUGR animals displayed decreased numbers of axonal tracts within the caudate nucleus (E), though mean axonal size was unaffected (F). Scale Bar = 1000 μm;*p<0.05; **p<0.01; ***p<0.001.

We next investigated the caudate nucleus, which has a large number of descending neuromotor fibre tracts and plays a key role in motor function and integration. Upon light microscopic investigation of LFB/H&E-stained sections (Fig 5A–5C), we observed a decrease in the area of the caudate nucleus in IUGR animals when compared to their Sham counterparts (Fig 5D). There was a trend of the caudate nucleus size to inversely correlate with the extent of growth restriction, however, this did not reach significance among IUGR groups. When fibre tracts were quantified manually using NF200+/MAG+ stained axons, there were significantly less tract counts in both IUGR groups when compared to Sham animals (Fig 5E). Lastly, we investigated mean tract size in the caudate nucleus. Here, we found no differences among groups (Fig 5F).

### Effects of IUGR on other sub-cortical structures

We then proceeded to assess changes in to various other white and grey matter structures that form connections and process information in conjunction with the previously assessed regions. The spectrum of injury observed in the CC, hippocampi, and caudate nuclei was recapitulated in other structures.

**Fig 6:**
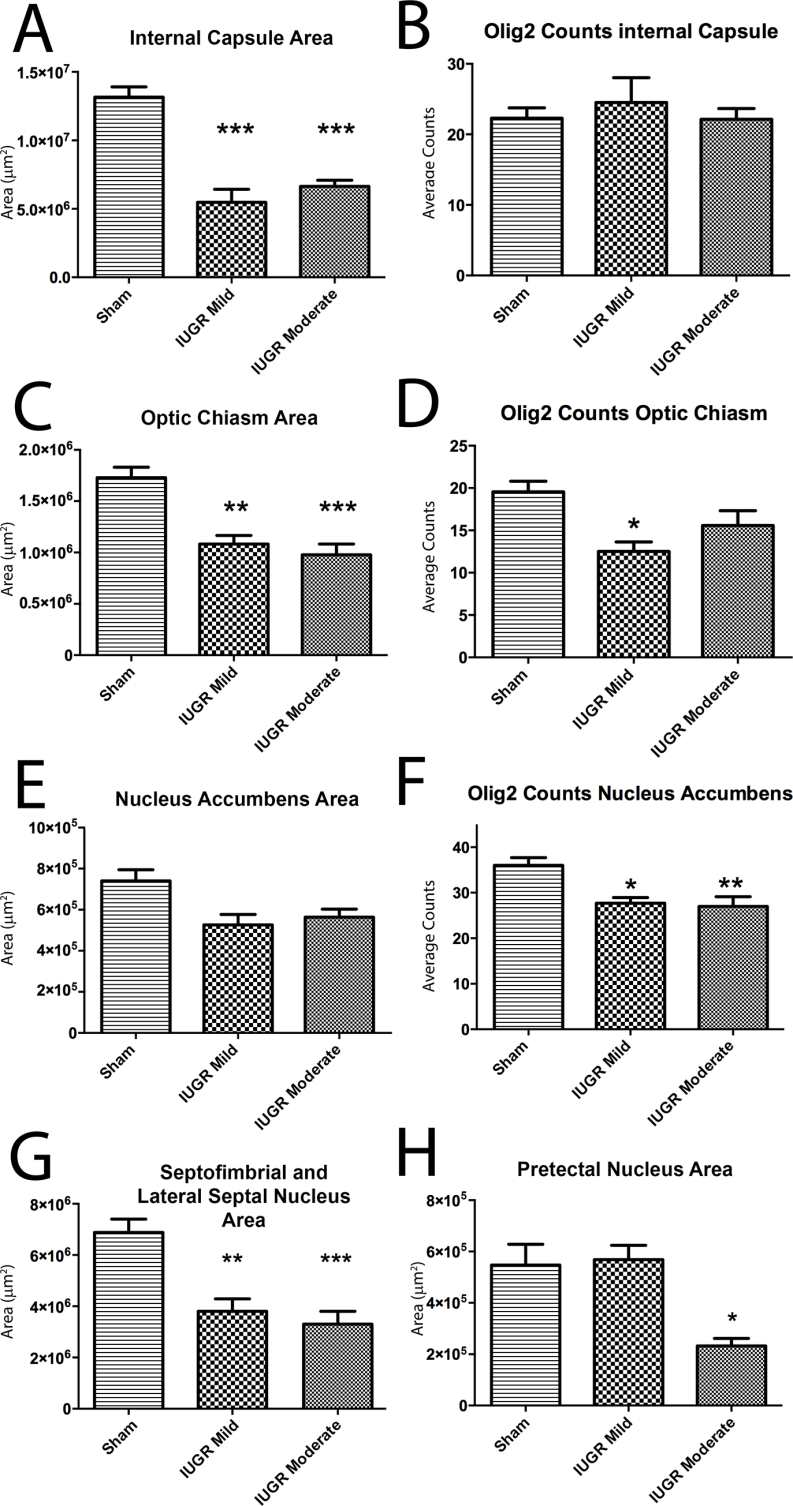
Structural abnormalities in sub-cortical structures of IUGR animals. Animals with IUGR displayed differences in sub-cortical structures when compared to Sham animals. Internal capsule size was reduced in both mild and moderate IUGR groups (A). The number of Olig2+ cells in the internal capsule remained unchanged (B). The optic chiasm showed similar results as the internal capsule (C), in addition to fewer OLs in the mildly injured group (D). There was no significant difference in the size of the nucleus accumbens between IUGR and sham animals (E). There were, however, fewer Olig2+ OLs in the nucleus accumbens of both mild and moderate IUGR animals when compared to sham animals (F). The septofimbrial and lateral septal nuclei were decreased in size in IUGR animals relative to sham controls (G). The pretectal nucleus was also significantly decreased in size in moderate IUGR animals only when compared to sham animals (H). *p<0.05; **p<0.01; ***p<0.001.

Both mild and moderate IUGR animals exhibited a decrease in the area of the internal capsule, an important white matter structure necessary for sensorimotor integration; however, there was no difference in Olig2+ cell counts among all groups (Fig 6A and 6B). IUGR-specific area loss was evident, when we investigated changes in the optic chiasm, which is a brain structure where the myelinated optic nerves partially cross (Fig 6C). Olig2+ cell counting revealed a decreased cell density in mildly, but not moderately, injured animals; although there was a trend toward reduced cell density in the latter group that did not reach significance (Fig 6D).

The nucleus accumbens, an important part of the striatal system, showed no significant reductions in area in both mild and moderate IUGR, but showed decreases in overall OL density in both groups of IUGR animals (Fig 6E and 6F). Interestingly, a decrease in area has also been observed in regions containing mainly unmyelinated axons. Therefore, Olig2+ cell counts could not be reliably performed. Septal nuclei showed reductions in area in both mild and moderate IUGR animals (Fig 6G). Pretectal nuclei were also reduced in size in the moderate IUGR group only when compared to Sham animals (Fig 6H). The mammillothalamic fasciculus showed no difference among groups in size (Sham vs. Mild p = 0.999, Sham vs. Moderate p = 0.820).

### Motor deficits on the Catwalk

**Fig 7:**
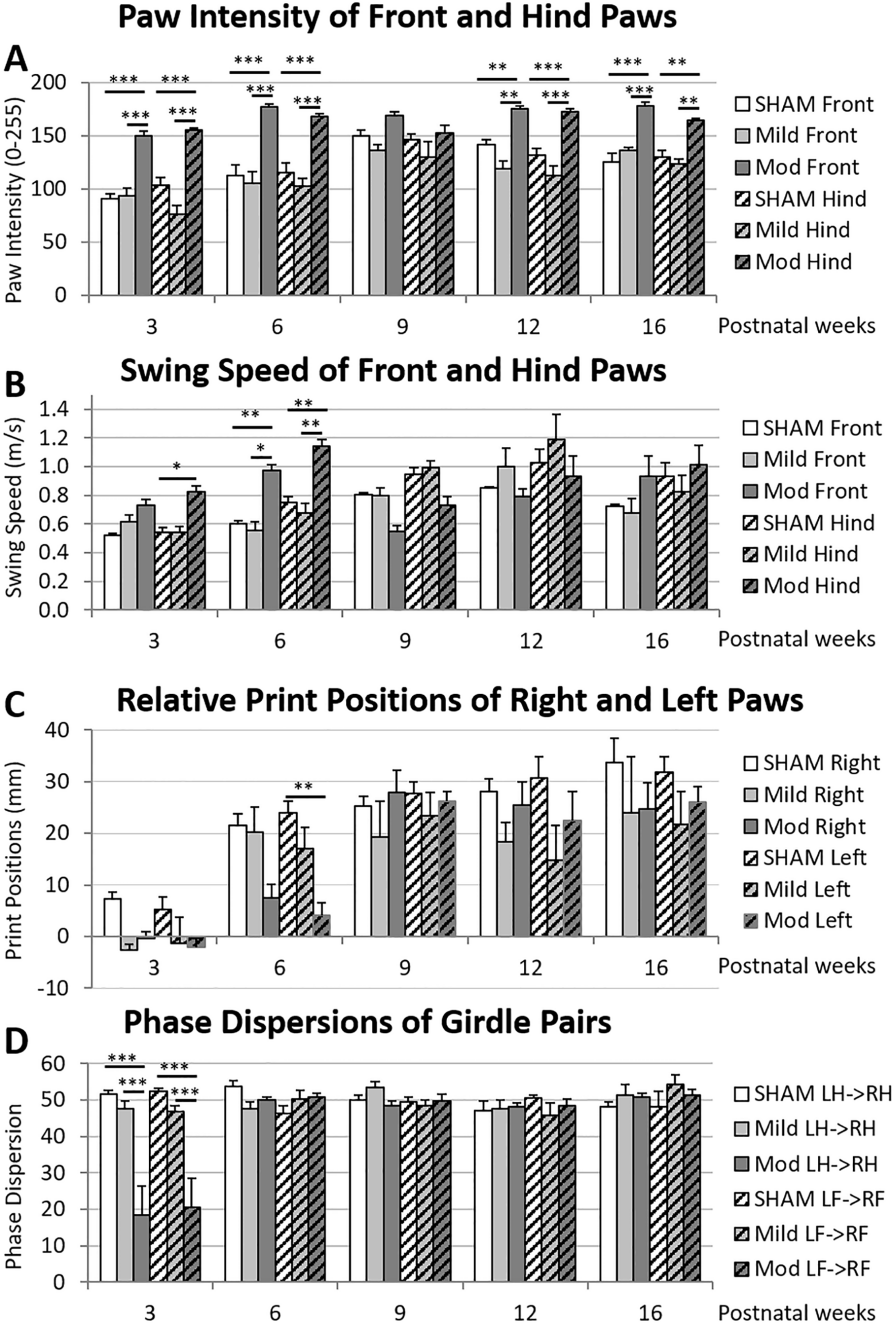
Motor gait deficits are present in IUGR rats. Animals with IUGR showed clear and consistent gait deficits at 3 weeks, some of which persisted into young adulthood. Paw intensity (0–255) for fore- and hindpaws was significantly increased in moderate IUGR animals (p<0.0001 for both) (A). Swing speed (m/s) for fore- and hindpaws did not show overall significance between IUGR and sham animals. There were, however, significant differences at early timepoints between moderate IUGR and sham animals (B). Relative print positions (mm) for right and left pairs of paws showed overall significant differences in the left paws of both mild and moderate IUGR animals when compared to sham animals (p = 0.0089 and p = 0.0019, respectively). There was a similar trend toward significance in the right paws of both mild and moderate IUGR groups (p = 0.0503 and p = 0.0603, respectively) (C). Phase dispersions (AU) of both fore and hind girdle paw pairs were significantly decreased in moderate IUGR animals when compared to sham animals (p = 0.0114 and p = 0.0002, respectively) (D). *p<0.05, **p<0.01, ***p<0.001.

To determine whether the observed anatomical abnormalities were reflected by behavioural deficits, we used the Catwalk Gait Analysis system (Fig 7). Various gait parameters were quantified, analyzed, and compared across groups. We saw a spectrum of deficits among groups, which recapitulates the heterogeneous human phenotype. Fig 7 presents some significant parameters.

#### Paw intensity

Front and hind paw intensities were significantly greater in the moderate IUGR group when compared to both mild IUGR and sham groups. Mild IUGR animals displayed greater hindpaw intensities than sham animals, but there was no difference in forepaw intensities between these two groups (Fig 7A). Interestingly these deficits in moderate IUGR animals persisted into adulthood (up to 16 weeks of age) and were independent of body weight, which normalized by 6 weeks of age.

#### Swing speed (m/s)

There was no overall significant difference in swing speed of the fore- and hindpaws between IUGR groups and sham animals. However, early timepoints did show significant differences mainly between moderate IUGR animals and sham animals, though these deficits resolved with age (Fig 7B).

#### Print positions

The relative positions of the left fore- and hindpaws to each other was significantly reduced in both groups of IUGR animals when compared to sham animals. The right paws showed a similar trend in both groups, though significance was not reached (Fig 7C).

#### Phase dispersion

There was a significant difference in phase dispersions of the fore- and hindpaw girdle pairs between moderate IUGR animals and sham animals. There was also a significant difference between moderate and mild IUGR animals in the phase dispersion of the hindpaw girdle pair. These differences were present at 3 weeks, but resolved with age (Fig 7D).

## Discussion

The work presented here investigates the role of PI via BUAL in a translational rodent model of CP. We investigated whether the IUGR rodent model could lead to defects that persist into adulthood and recapitulate the spectrum of phenotypes that exists in the clinic. Our study is the first to utilize a combination of standard laboratory techniques (histology) as well as clinically relevant outcome modalities (MRI, behavioural testing). Here we have characterized the adult phenotypic heterogeneity in this model, which recapitulates clinical presentations of the disorder. Furthermore, it is the first to describe the subtle anatomical differences within the brain that can be successfully reproduced in this rat IUGR model. This model provides a platform through which to assess future treatments for EoP and CP, and it could prove integral to the progression of the field.

Although the weights of all animals with IUGR normalized during the course of the study, structural brain abnormalities persisted in injured animals into adulthood. Furthermore, by taking advantage of the inherent variability in the IUGR injury model and postnatally categorizing animals into different injury-severity groups based on the extent of growth restriction at birth, we were able to reflect the variability of injury that is observed in the clinic. Histological analysis revealed changes to the size of various regions, as well as changes to specific cell populations, with more structural damage in adulthood positively correlating with the extent of growth restriction at birth.

Here, we showed deficits in adult brain area, which reached significance in moderately injured animals but not mildly injured animals. This could be due to differential acute and/or chronic injury kinetics. Children born with fetal growth restriction often exhibit concomitant learning and cognitive impairment. There is emerging literature to suggest subtle anatomical differences in these cohorts both clinically [43,44] and preclinically [32].

The assessment of WM tracts was an integral part of this study, as EoP and CP caused by fetal ischemic brain damage often lead to impairments in OL function and subsequent neural processing. OL precursors have been shown to be particularly susceptible to hypoxic injury [16,45,46], and in our rat model of IUGR, we demonstrate this effect on WM structures through concomitant reduction in both white matter area and Olig2+ OL population. This is consistent with findings in other PI models in rats [35,47,48] and guinea pigs [32,49,50]. We also demonstrate that these deficits in myelination persist into adulthood. When we investigated myelination patterns in the CC, we found that moderately injured animals possessed fewer OLs and axons, but normal amounts of myelination within other white matter systems. Interestingly, moderately injured animals displayed a higher number of unmyelinated axons than mild IUGR animals in the CC, suggesting that this IUGR model begins to specifically target white matter in the CC as the severity of injury increases. While a major challenge to studying preterm and neonatal brain damage in preclinical models is the ability of the rodent brain to mimic the human phenotype, the persistent and widespread WM injury in our IUGR model suggests that it is a robust model for testing translational therapies, especially in the context of repairing injured WM.

CP is a heterogeneous disorder and many patients present with a wide array of clinical phenotypes. Both the internal capsule and basal ganglia can be affected in these patients, leading to various motor deficits. In line with the clinical picture of CP, the caudate nucleus was smaller in IUGR animals, accompanied by fewer, yet typically-sized axonal tracts. Interestingly, the internal capsule was smaller in IUGR groups compared to controls, without accompanying decreases in OL numbers within the tracts. In characterizing this model of IUGR, we sought to evaluate the integrity of various other brain structures, which could be used for assessment of the efficacy of therapeutic interventions in future studies. Similar to the internal capsule, the optic chiasm and the septofimbrial nucleus/lateral septal nucleus were smaller in IUGR groups compared to controls, without decreases in OL numbers (with the exception of the optic chiasm, which showed significantly decreased cell counts in mild IUGR animals only). Pretectal nuclei were smaller only in moderately injured IUGR animals. In contrast to these results, the nucleus accumbens showed no significant differences in size among groups, though Olig2+ cell counts were lower in both IUGR groups. Interestingly, even in a model of “mild intrauterine hypo-perfusion”, neurological development impairment is observed, with reduced white and gray matter areas despite evident tissue injury [51].

Gray matter injury is confirmed by the neuronal cell loss observed in areas associated with learning and cognition, such as the hippocampus. There was also a trend of decreasing overall size of the hippocampus, which corresponds to the human phenotype [43]. This reached significance only in moderately affected animals. When we looked more closely, we observed concomitant reductions in neuronal density and thickness of the CA2 region in both moderate and mild IUGR animals, as well as deficits in the CA3 region and dentate gyrus in moderately injured animals only. These results are in line with previous studies that have found neuronal cell death and degenerating neurons within the hippocampus in neonates [21,52]. To our knowledge, this work is the first to demonstrate a persistent, long-term reduction in neuronal density following BUAL in rats, and provides a preclinical platform for testing therapeutic interventions for EoP or CP.

Regarding motor function, the gait analysis revealed abnormal and persistent deficits in paw intensity in moderate IUGR animals. Additional deficits in swing speed, print position (moderate only) and phase dispersion were present in mild and moderate IUGR animals, but resolved during development, which could suggest some plasticity mechanisms leading to spontaneous recovery. This is similar to findings of mild deficits in gait and posture in unilaterally injured rats [35]. More importantly, these deficits mimic the human spastic disease phenotype [53], which exhibits many of these markers, some resolving during the course of maturation. The catwalk is a sensitive measurement to changes in gait and has been used widely in various models of brain [54] and spinal cord [55] injury. The results presented in this IUGR model suggest that the use of additional behavioural tests for assessing the efficacy of therapeutic interventions in the long-term may be needed, as many, though not all, of the gait parameters became comparable to controls over time.

The myelination impairment is shown by early changes in axial and radial diffusivities in the CC, Ec, and Ca reported here, which correlate well with the later regional deficiencies of WM and OL density. This result is indicative of early degeneration of axonal and myelin integrity in these regions as a consequence of IUGR. While global reductions in FA have been reported in the acute phase after injury in both animal [56,57] and human data [58,59] including IUGR [60], the pseudo-normalization of FA in the sub-acute phase after injury is well documented [61]. Developmentally, FA alone may be insufficient to explain complex regional pathology. The global increase in radial diffusivity presented here is suggestive of a lack of global fibre directionality due to myelin injury [62]. Pixel by pixel analysis of MRI and histological markers demonstrate relationships between axial diffusivity, axonal injury, and behaviour in several injury models [62–65]. While normalizing MRI and histological images for pixel wide analysis was not possible here, the regional changes in DWI are consistent with the histological and behavioural deficits. A greater delineation of the extent of regional myelin injury in IUGR could be achieved by using additional MR techniques such as 3D protocol, T2 mapping, magnetization transfer (MT), myelin water imaging [66,67] and quantitative WM fiber tracking algorithms [68].

In conclusion, our findings support the use of BUAL in the rat as a robust and translationally relevant model for IUGR, with which multimodal parameters can be used to study longitudinal change in disease progression and therapeutic interventions. We show for the first time that variance in the level of IUGR severity can recapitulate various aspects of clinical disease, which will be useful when developing translational treatment strategies. Indeed, human studies have demonstrated that placental insufficiency is the most prevalent cause of growth restriction in developed countries [21], contributing to a large proportion of the clinical EoP and CP phenotype. Utilizing this model in combination with longitudinal, multi-faceted assessment tools of pathology and behaviour, we will be able to design future experiments to address key clinical features across the disease spectrum, thereby tailoring treatment interventions to tackle the heterogeneous nature of brain damage in the developing central nervous system.

## Supporting information

S1 Fig Diagram of IUGR surgery. This figure illustrates where the ligations of the uterine arteries were performed in order to induce PI and subsequent IUGR.
(PPTX)

S2 Fig Diagram of areas examined for immunohistochemical quantification. This figure illustrates the five areas examined in the CC (green) and the four areas examined in the hippocampus (yellow), for the purpose of cell counting.
(TIF)

S3 Fig Diagram of thickness measurements. This figure illustrates the three thickness measurements taken in the CC (green) and the six thickness measurements taken in the hippocampus (yellow).
(TIF)

S1 Table Mild IUGR MRI data. At 3 weeks of age, abnormalities in axial diffusivity in the corpus callosum were evident in mild IUGR animals. Tabulated DTI metrics across the sham and mild IUGR cohorts are presented. Locations of significant differences (gCC and sCC) are highlighted by * (p<0.05).
(DOCX)
