# Supplementary figures and images for: The extent of intrauterine growth restriction determines the severity of cerebral injury and neurobehavioural deficits in rodents
Source: PLoS One. 2017 Sep 21;12(9):e0184653. doi: 10.1371/journal.pone.0184653 (PMC5608203; doi:10.1371/journal.pone.0184653)

## Slide 1
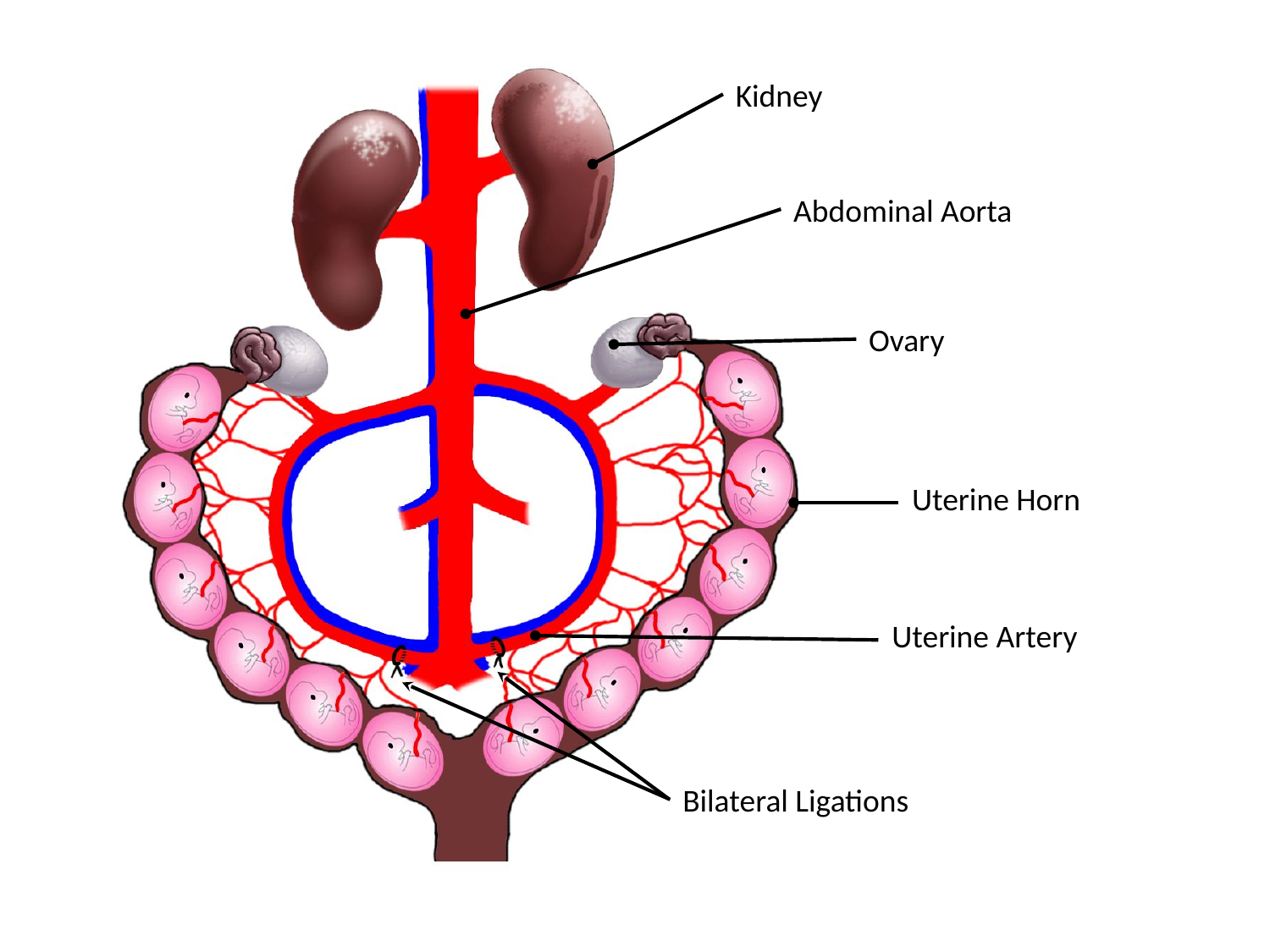

Kidney
Abdominal Aorta
Ovary
Uterine Horn
Uterine Artery
Bilateral Ligations

Supplement: S1 Fig — This figure illustrates where the ligations of the uterine arteries were performed in order to induce PI and subsequent IUGR. (PPTX) [file pone.0184653.s001.pptx]

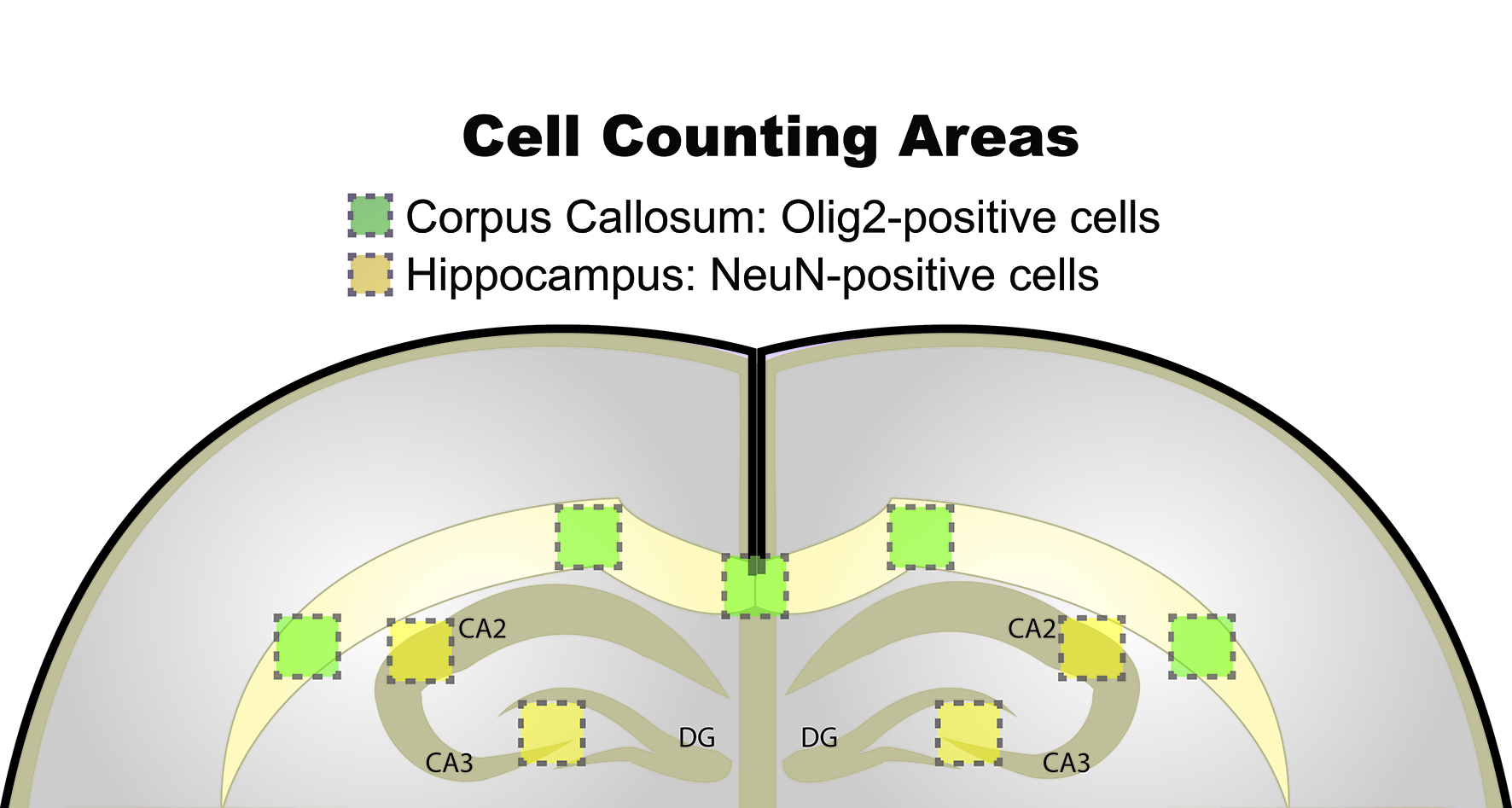

Supplement: S2 Fig — This figure illustrates the five areas examined in the CC (green) and the four areas examined in the hippocampus (yellow), for the purpose of cell counting. (TIF) [file pone.0184653.s002.tif]

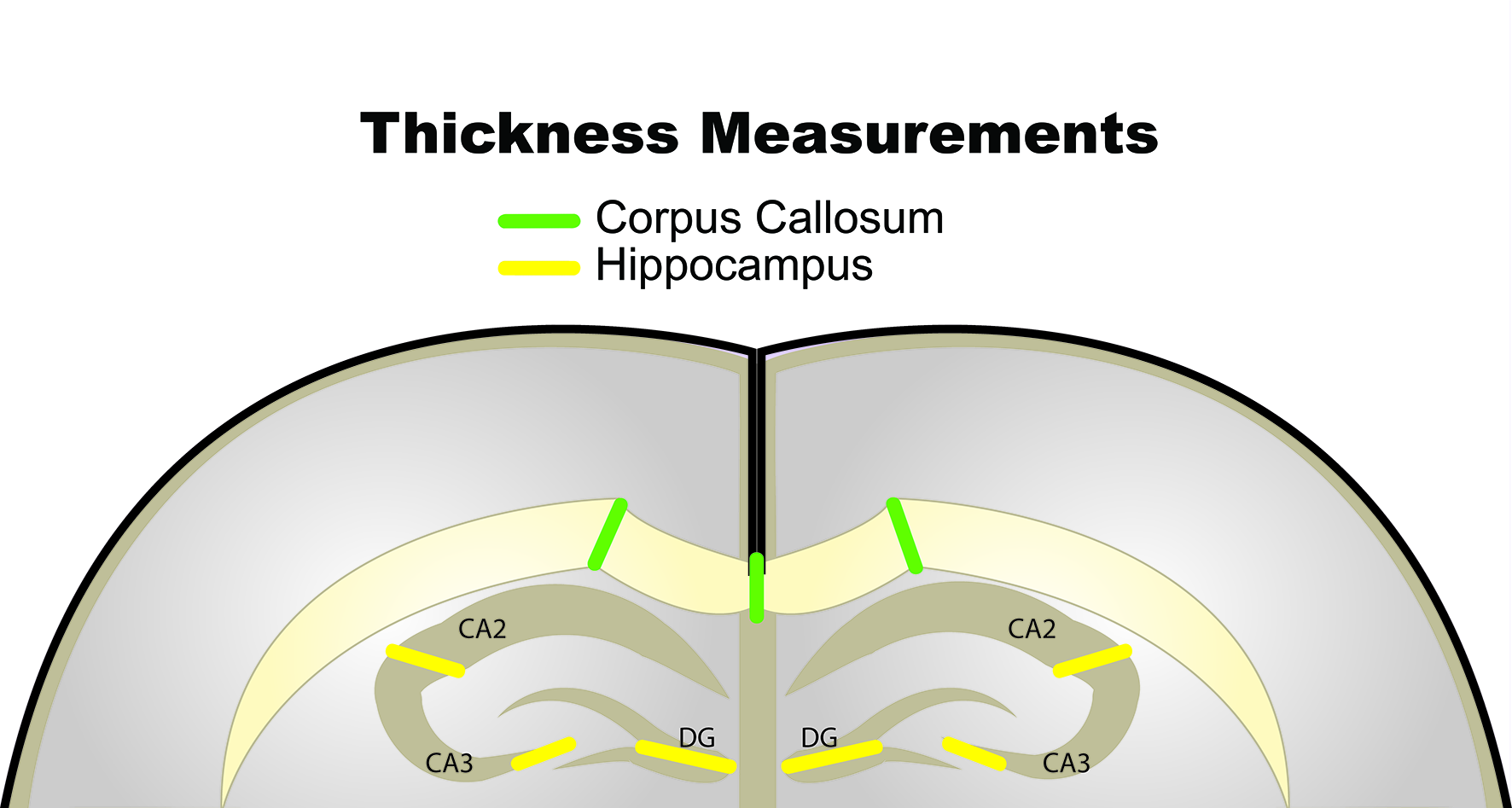

Supplement: S3 Fig — This figure illustrates the three thickness measurements taken in the CC (green) and the six thickness measurements taken in the hippocampus (yellow). (TIF) [file pone.0184653.s003.tif]
