# Supplementary material for: The extent of intrauterine growth restriction determines the severity of cerebral injury and neurobehavioural deficits in rodents
Source: PLoS One. 2017 Sep 21;12(9):e0184653. doi: 10.1371/journal.pone.0184653 (PMC5608203; doi:10.1371/journal.pone.0184653)
Supplement: S1 Table — At 3 weeks of age, abnormalities in axial diffusivity in the corpus callosum were evident in mild IUGR animals. Tabulated DTI metrics across the sham and mild IUGR cohorts are presented. Locations of significant differences (gCC and sCC) are highlighted by * (p<0.05). (DOCX) [file pone.0184653.s004.docx]

**S1 Table Mild IUGR MRI data.** At 3 weeks of age, abnormalities in axial diffusivity in the corpus callosum were evident in mild IUGR animals. Tabulated DTI metrics across the sham and mild IUGR cohorts are presented. Locations of significant differences (gCC and sCC) are highlighted by * (p<0.05).

|  | **SHAM** | | | | | | **MILD** | | | | | |
| --- | --- | --- | --- | --- | --- | --- | --- | --- | --- | --- | --- | --- |
|  | **FA** | | **Axial (x10-3)** | | **Radial (x10-3)** | | **FA** | | **Axial (x10^-3^)** | | **Radial (x10^-3^)** | |
| **ROI** | **Mean** | **SEM** | **Mean** | **SEM** | **Mean** | **SEM** | **Mean** | **SEM** | **Mean** | **SEM** | **Mean** | **SEM** |
| **gCC** | 0.715 | 0.005 | 1.695 | 0.015 | 0.425 | 0.020 | 0.69 | 0.03 | 1.83* | 0.03 | 0.40 | 0.02 |
| **gEc** | 0.485 | 0.010 | 1.225 | 0.020 | 0.595 | 0.015 | 0.43 | 0.01 | 1.19 | 0.02 | 0.67 | 0.04 |
| **gCa** | 0.275 | 0.020 | 1.045 | 0.015 | 0.695 | 0.015 | 0.22 | 0.02 | 1.17 | 0.08 | 0.80 | 0.06 |
| **bCC** | 0.575 | 0.005 | 1.530 | 0.032 | 0.575 | 0.016 | 0.56 | 0.02 | 1.70 | 0.12 | 0.64 | 0.04 |
| **bEc** | 0.585 | 0.005 | 1.500 | 0.034 | 0.535 | 0.022 | 0.50 | 0.01 | 1.48 | 0.08 | 0.63 | 0.02 |
| **bCa** | 0.230 | 0.005 | 1.060 | 0.027 | 0.745 | 0.011 | 0.24 | 0.01 | 1.12 | 0.07 | 0.80 | 0.06 |
| **sCC** | 0.515 | 0.005 | 1.455 | 0.021 | 0.650 | 0.018 | 0.53 | 0.01 | 1.63* | 0.09 | 0.68 | 0.05 |
| **sEc** | 0.540 | 0.005 | 1.430 | 0.028 | 0.570 | 0.021 | 0.54 | 0.02 | 1.49 | 0.09 | 0.63 | 0.02 |
| **sCa** | 0.230 | 0.005 | 1.070 | 0.021 | 0.775 | 0.011 | 0.22 | 0.01 | 1.17 | 0.10 | 0.81 | 0.05 |
